# Investigating the association between serum human papillomavirus type 16 E7 antibodies and risk of head and neck cancer

**DOI:** 10.1002/cam4.3944

**Published:** 2021-05-04

**Authors:** Cheng‐Chih Huang, Yu‐Chu Su, Chan‐Chi Chang, Wei‐Ting Lee, Chun‐Yen Ou, Yuan‐Hua Wu, Shang‐Yin Wu, Yu‐Hsuan Lai, Jehn‐Shyun Huang, Ken‐Chung Chen, Wei‐Ting Hsueh, Sen‐Tien Tsai, Chia‐Jui Yen, Jang‐Yang Chang, Mei‐Ling Tsai, Chen‐Lin Lin, Ya‐Ling Weng, Han‐Chien Yang, Yu‐Shan Chen, Jenn‐Ren Hsiao, Jeffrey S. Chang

**Affiliations:** ^1^ Department of Otolaryngology College of Medicine National Cheng Kung University Hospital National Cheng Kung University Tainan Taiwan; ^2^ Clinical Medicine Research Center College of Medicine National Cheng Kung University Hospital National Cheng Kung University Tainan Taiwan; ^3^ Institute of Clinical Medicine College of Medicine National Cheng Kung University Tainan Taiwan; ^4^ Department of Radiation Oncology College of Medicine National Cheng Kung University Hospital National Cheng Kung University Tainan Taiwan; ^5^ Division of Hematology/Oncology Department of Internal Medicine College of Medicine National Cheng Kung University Hospital National Cheng Kung University Tainan Taiwan; ^6^ Department of Stomatology College of Medicine National Cheng Kung University Hospital National Cheng Kung University Tainan Taiwan; ^7^ Institute of Biotechnology and Pharmaceutical Research National Health Research Institutes Zhunan Town Miaoli Taiwan; ^8^ Department of Nursing College of Medicine National Cheng Kung University Hospital National Cheng Kung University Tainan Taiwan; ^9^ National Institute of Cancer Research National Health Research Institutes Tainan Taiwan

**Keywords:** cancer prevention, cancer risk factors, epidemiology, head and neck cancer

## Abstract

Human papillomavirus (HPV) is recognized as a major cause of oropharyngeal cancer (OPC) in Western countries. Less is known regarding its contribution to the OPC occurring in Asia. The current study aimed to investigate the association between antibody responses to HPV16 E7 and head and neck cancer (HNC) risk in a hospital‐based case–control study conducted in Taiwan with 693 HNC cases and 1,035 controls. A positive association was observed between seropositivity to HPV16 E7 and OPC risk, whereas no significant association was found in the non‐OPC cases. The increased OPC risk associated with seropositivity to HPV16 E7 was more significant among nonbetel quid or noncigarette users. Seropositivity to HPV16 E7 showed moderate agreement with P16 expression in OPC. OPC patients that were seropositive to HPV16 E7 or p16 positive were more highly educated and less likely to use alcohol, betel quids, and cigarettes compared to HPV16 E7 seronegative or p16 negative OPC patients. Furthermore, patients with p16 positive OPC were more likely to be women compared to patients with p16 negative OPC, likely owing to the low prevalence of alcohol, betel quid, and cigarette users among women. Overall, this study suggested that similar to Western countries, HPV may also be an important risk factor of OPC in Taiwan. With the declining consumption of betel quids and cigarettes in Taiwan, a higher percentage of OPC cases in Taiwan will be attributed to HPV in the future. Public health measures, including HPV vaccination, need to be implemented to prevent the occurrence of HPV‐positive OPC.

## Abstract 2

The current study found a a positive association between seropositivity to HPV16 E7 and oropharyngeal cancer risk. This study suggested that similar to Western countries, HPV may also be an important risk factor of OPC in Taiwan.

## 1 BACKGROUND

Cancers arising from the oral cavity, oropharynx, hypopharynx, and larynx are together known as head and neck cancer (HNC), which is the seventh most common cancer in the world, with 710,000 new cases diagnosed worldwide each year.^1^ Strong evidence from previous studies has established alcohol drinking, betel quid chewing, and cigarette smoking as the major risk factors of HNC.^2^ Besides the use of alcohol, betel quids, and cigarettes, a substantial proportion of oropharyngeal cancers (OPCs) are attributed to human papillomavirus (HPV).

The incidence of OPC has been rising, especially in Western countries. Van dyne et al. reported that from 1999 to 2015, OPC surpassed cervical cancer to become the most common HPV‐associated cancer in the United States, with the incidence of OPC showing an increase of 2.7% per year among men and 0.8% per year among women.^3^ Chaturvedi et al. reported a 225% increase in the incidence of HPV‐positive OPC in the United States from 1988 to 2004, whereas the incidence of HPV‐negative OPC decreased by 50% during the same period.^4^ In Canada, although the overall incidence of HNC showed a significant decrease from 1992 to 1998 and remained stable from 1998 to 2009, the incidence of HPV‐associated OPC rose significantly during the same period with an annual percentage change of 2.7.^5^ In Eastern Denmark, 62% of OPC cases were HPV‐positive.^6^ In addition, the increase in the incidence of OPC from 4.0 per 100,000 in 2011 to 4.5 per 100,000 in 2014 was mainly driven by the increase in HPV‐positive cases.^6^ A study from Germany showed that the prevalence of HPV‐positive OPC increased from 28% in 2004–2006 to 59% in 2012–2013 and the increase in the incidence of OPC in this population was largely due to the increase in HPV‐positive cases.^7^ The incidence trends of HPV‐associated HNC have not been well documented in Asian countries. Previously, we used data from the Taiwan Cancer Registry and categorized HNC into HPV‐related and HPV‐unrelated according to the anatomical site.^8^ We found that the incidence of HPV‐related HNC (mainly tonsil and the base of the tongue) in Taiwan increased from 1.3 per 100,000 in 1995 to 3.3 in 2009 with an annual percentage change of 6.9.^8^ This suggested that HPV may also contribute to a significant proportion of OPCs occurring in Taiwan.

HPV16 is the main HPV type that promotes the development of HPV‐positive OPC.^9^ E6 and E7 are two oncoproteins generated upon the integration of HPV DNA into the genome of the human host.^10^ E6 promotes the degradation of p53, which is a key protein in DNA damage response, thereby impairing its function.^10^ The binding of E7 to pRB inactivates pRB, resulting in the dysregulation of the normal cell cycle that ultimately leads to malignant cell transformation.^10^ Thus, antibodies to HPV16 E6 and E7 have been considered as useful serological biomarkers for the oncogenesis of HPV‐associated HNC. A nested case–control study from the United States reported that 43% of OPC cases were seropositive for HPV16 E6 and the antibodies could be detected as early as 13 years before the diagnosis of OPC.^11^ The 10‐year cumulative risk of OPC among individuals who tested seropositive for HPV16 E6 was 6.2% for men and 1.3% for women, whereas individuals who were seronegative for HPV16 E6 had a low 10‐year cumulative risk of 0.04% for developing OPC.^11^

Many studies, mostly from Western countries, have investigated the association between antibody responses to HPV and HNC risk^11^, ^12^, ^13^, ^14^, ^15^, ^16^, ^17^, ^18^, ^19^, ^20^, ^21^, ^22^, ^23^ ; however, only one study has been conducted in Asia,^18^ had a small sample size with only 50 oral cancer cases and 50 controls and did not include OPC, which is the site of HNC most closely associated with HPV.^18^ The current study aimed to investigate the association between antibody responses to HPV16 E7 and HNC risk in a study population of 693 HNC cases and 1035 controls to evaluate the potential contribution of HPV to the development of HNC occurring in Taiwan. The results generated by this study can be used for cross‐continent comparison with results of the studies conducted in the Western populations to determine the global pervasiveness of HPV infection in the occurrence of HNC.

## 2 MATERIALS AND METHODS

The design and the contents of the current study were reviewed and approved by the institutional review boards of the National Health Research Institutes and the National Cheng Kung University Hospital. Each potential study participant received a detailed explanation of the study from a trained interviewer. Individuals who agreed to join the study were then asked to provide signed informed consent.

### 2.1 Study subject recruitment

The HNC cases were recruited by an ongoing HNC case–control study that commenced participant recruitment on 1 September 2010 in the Department of Otolaryngology and the Department of Stomatology at the National Cheng Kung University Hospital. Eligible subjects for the current study were patients diagnosed with pathologically confirmed squamous cell carcinoma of the oral cavity, oropharynx, hypopharynx, and larynx. Control subjects age and gender frequency matched to the cases were recruited as reference for comparison and included patients who received surgery for noncancerous conditions unrelated to the consumption of alcohol, betel quids, and cigarettes. The recruitment of the cases and controls was conducted by frequency‐matching according to the distribution of 12 gender × age (divided into six 10‐year strata from age 20 to 80) groups to produce similar distributions of gender and age between the cases and controls. Due to the ongoing recruitment process, there might have been some slight imbalances in the distributions of age and gender between the cases and controls. These imbalances were adjusted by including gender and age as covariates in the statistical analyses. Other eligibility criteria for both the cases and controls included as follows: (a) no previous diagnosis of cancer, (b) aged between 20 and 80, and (c) the ability to understand the purpose and the contents of the study and provide informed consent. The current analysis included subjects recruited from 1 September 2010 to 22 July 2019.

### 2.2 Data collection using in‐person interview

An in‐person interview was conducted with each study participant by a trained interviewer to collect information on gender, age, education, income, and consumption of alcohol, betel quids, and cigarettes. These variables were included in the multivariable logistic regression models as potential confounders to assess the independent association between serum antibodies to HPV16 and HNC risk.

### 2.3 Blood sample collection and processing

A red top vacutainer was used to obtain blood sample from each case or control subject before receiving any treatment (surgery, radiation therapy, or chemotherapy for the cases and surgery for the controls), medications, or intravenous fluid. The vacutainer was centrifuged to collect the serum. The serum was then stored in an Eppendorf tube and placed in the −80°C refrigerator until ready to use.

### 2.4 Confirmation of HPV E6 and E7 proteins with Western Blot

Histidine‐tagged, recombinant HPV16 E6 and E7 proteins (his‐tag HPV16 E6, E7), which were expressed in *Escherichia coli* and purified under native condition, were obtained from ProteinX Lab (San Diego, CA, USA). Western blot was used to confirm the identity of these proteins (Figure S1). Briefly, 50 ng HPV16 E6 and 100 ng HPV16 E7 proteins were loaded to 15% sodium dodecyl sulfate polyacrylamide gel electrophoresis (SDS‐PAGE) under reducing conditions. A rabbit polyclonal anti‐HPV16 E6 antibody (1/10000) (ABIN 2918957, antibodies‐online GmbH, Aachen, Germany) and a mouse monoclonal antibody (1/500) (clone ED17, Santa Cruz, Dallas, Texas, USA) were used to detect HPV16 E6 and E7 proteins, respectively. Horseradish peroxidase (HRP)‐conjugated, goat anti‐rabbit (0.2 g/mL) (#A27036, Thermo Scientific, Rockford, lL, USA) and goat anti‐mouse IgG (0.2 g/mL) (#A28177, Thermo Scientific) were used as secondary antibodies. Specific signals were then developed using the chemoilluminescence method on a Kodak BioMax ML film.

### 2.5 Detection of human anti‐HPV16 E7 IgG with enzyme‐linked immunosorbent assay (ELISA)

To detect human serological anti‐HPV16 E6 and E7 immunoglobulin G (IgG), we first tried to establish ELISA tests for the quantification of serological IgGs against HPV 16 E6 and E7 proteins. However, because a reliable ELISA test against HPV16 E6 could not be successfully established, only the serological responses to HPV16 E7 are reported in the current study. Briefly, HPV16 E7 proteins were first diluted in carbonate buffer (15 mM carbonate‐35 mM bicarbonate, pH 9.6) and were coated on each well (100 ng in 100 μL/well) of 96‐well plates (# 467320, Nunc, Thermo Scientific). The plates were incubated at 4℃ overnight. After phosphate‐buffered saline (PBS, PH 7.2) wash (5 min at room temperature for three times), the coated wells were blocked with 300 μL PBS‐1% bovine serum albumin (BSA) at room temperature for 60 min, followed by a 5‐min wash with 300 μL PBS‐0.05% Tween 20 (PBS‐T) at room temperature (RT) for three times. The wells were then incubated with individual human serum samples (diluted in PBS‐1%BSA, 1:50) at room temperature for 60 min. After washing with PBS‐T, human IgGs specific for HPV16 E7 proteins were detected with an HRP‐conjugated, goat anti‐human IgG antibody (0.2 μg/mL) (#A18811, Thermo Scientific). After washing, ABTS [2, 2'‐azino‐bis(3‐ethyl‐ benzthiazoline‐6‐sulphonic acid)] (100 μL/well) (#34026, Thermo Scientific) was added to each well and incubated at room temperature for 10 min. Following incubation, the color development was stopped in each well by adding 1% sodium dodecyl sulfate (SDS). The optical density (OD) was detected at a wavelength of 405 nm by using a spectrophotometer. To minimize operational variations during experiments, a high throughput multifunctional robotic platform (Freedom Evo 200, Tecan, Switzerland) was optimized and used in conducting the ELISA procedure. The serum sample of each subject was analyzed in duplicate on the same plate. A corrected OD was generated by averaging the two ODs and subtracting the OD of the blank control from the average. The intraassay % coefficient of variability (CV) and the interassay % CV were 8.2% and 11.9%, respectively.

### 2.6 P16 immunohistochemistry (IHC) staining of oropharyngeal tumor tissue

Paraffin‐embedded tumor tissue blocks were available for 71 of the 105 OPC cases (67.6%) for determining the HPV status of OPC by using p16 IHC staining. The 71 OPC cases with and the 35 OPC cases without paraffin‐embedded tumor tissue blocks had a similar percentage of women (9/71 or 12.7% vs 4/34 or 11.8%, Fisher's exact test, *p* = 1.00). Briefly, for p16 IHC staining, 4‐µm thickness sections were first obtained from archival formalin‐fixed, paraffin‐embedded tissue blocks. The slides were then deparaffinized and rehydrated through graded alcohols, followed by using immunohistochemical detection of p16 protein expression using an in vitro diagnostic CINtec® p16 Histology kit (mtm laboratories AG, Heidelberg, Germany) containing a prediluted mouse monoclonal antibody specific against p16 (clone E6H4). Each specific section was considered “positive for p16 expression” when strong and diffuse nuclear and cytoplasmic staining was observed in more than 70% of the tumor cells.^24^

### 2.7 Statistical analysis

To compare the differences in the distributions of gender, age, education, income, alcohol drinking, betel quid chewing, and cigarette smoking, chi‐squared tests were performed for categorical variables and *t*‐tests were used for continuous variables.

Multivariable unconditional logistic regression was performed to calculate the odds ratio (OR) and 95% confidence interval (CI) to examine the association between HPV16 E7 serostatus and HNC risk, adjusted for gender, age, education, income, and use of alcohol, betel quids, and cigarettes. The mean OD value of the E7 antibody plus three standard deviations among the controls was defined as the cutoff value for being seropositive to E7. The analysis was first performed with all HNC cases and then separately for OPC and non‐OPC (cancers of the oral cavity, hypopharynx, and larynx) cases.

The association between seropositivity to HPV16 E7 and HNC risk was further analyzed stratified by the use of alcohol, betel quids, and cigarettes to evaluate whether the association might differ according to the status of these lifestyle behaviors. The heterogeneity between the strata of these lifestyle behaviors was evaluated by including a product term (antibody status for HPV16 E7 × use of alcohol, betel quids, or cigarettes) in the unconditional logistic regression model. The significance of heterogeneity was assessed by the log‐likelihood ratio test comparing the regression model with the product term to the model without the product term.

Wilcoxon‐rank sum tests, chi‐square tests, or Fisher's exact tests were performed to compare the distributions of demographic and lifestyle characteristics between HPV16 E7 antibody positive and negative HNC patients. The agreement between the serostatus of HPV16 E7 and the p16 status of the OPC tumor tissue was assessed using the kappa statistic. Finally, *t*‐tests, chi‐square tests, or Fisher's exact tests were performed to compare the distributions of demographic characteristics and lifestyle behaviors between patients with p16‐positive and p16‐negative OPC.

## 3 RESULTS

Figure S2 presents the flowchart for selecting subjects for the current analysis. During 1 September 2010–22 July 2019, we identified 1,533 eligible HNC cases and 1,720 eligible controls. A total of 1,126 eligible HNC cases (178 missed cases and 229 refusals, participating percentage = 74%) and 1,424 eligible controls (111 missed controls and 185 refusals, participating percentage = 83%) agreed to participate in the study. The missed cases and controls were those who we missed to contact with before they received any treatment (surgery, radiation therapy, or chemotherapy for the cases and surgery for the controls) due to scheduling, and thus pretreatment blood samples could not be collected. In addition, we decided not to interview after treatment because the majority of the HNC patients have difficulty while answering questions after surgery. Those who did not participate in this study were younger but had a similar distribution of gender compared to those that participated in the study (Supplementary Table 1). The current analysis included 693 of the 1,126 HNC cases (62%) and 1,035 of the 1,424 controls (73%) with previously unthawed serum samples available for measuring serum antibodies to HPV16. Those with and without unthawed serum samples were similar in the distributions of age, gender, education, and use of alcohol, betel quids, and cigarettes (Table S2). The 693 HNC cases in the current analysis included 640 men (92.4%) and 53 women (7.6%). Among the 693 cases, 105 (15.2%) were located in the oropharynx, and 588 (84.8%) were located in other sites (oral cavity, hypopharynx, and larynx). Table S3 presents the distribution of the clinical diagnoses for the 1035 controls included in the current study.

**TABLE 1:** Demographic and lifestyle characteristics of the head and neck cancer patients and controls

Characteristics	Overall			Men			Women		
	Case N = 693 *n* (%)	Control N = 1035 n (%)	*p* ^a^	Case N = 640 n (%)	Control N = 992 n (%)	*p* ^a^	Case N = 53 n (%)	Control N = 43 n (%)	*p* ^a^
Age (years)									
Mean (SE)	55.6 (0.4)	54.7 (0.3)	0.081	55.3 (0.4)	54.4 (0.3)	0.109	59.6 (1.8)	61.0 (1.9)	0.609
Gender									
Men	640 (92.4)	992 (95.9)	0.002						
Women	53 (7.6)	43 (4.1)							
Education									
≦Elementary school	180 (26.0)	157 (15.2)	<0.001	164 (25.6)	137 (13.8)	<0.001	16 (30.2)	20 (46.5)	0.043
Junior high	208 (30.0)	163 (15.7)		195 (30.5)	161 (16.2)		13 (24.5)	2 (4.6)	
High school/Technical school	230 (33.2)	366 (35.4)		215 (33.6)	351 (35.4)		15 (28.3)	15 (34.9)	
Some college or more	75 (10.8)	349 (33.7)		66 (10.3)	343 (34.6)		9 (17.0)	6 (14.0)	
Alcohol drinking									
Never +Occasional	225 (32.5)	608 (58.7)	<0.001^b^	178 (27.8)	566 (57.1)	<0.001^b^	47 (88.7)	42 (97.7)	0.235
Former regular	103 (14.9)	98 (9.5)		101 (15.8)	97 (9.8)		2 (3.8)	1 (2.3)	
Current regular	364 (52.5)	329 (31.8)		360 (56.2)	329 (33.2)		4 (7.5)	0 (0.0)	
Unknown	1 (0.1)	0 (0.0)		1 (0.2)	0 (0.0)		0 (0.0)	0 (0.0)	
Betel quid chewing									
Never	186 (26.8)	742 (71.7)	<0.001^b^	137 (21.4)	699 (70.5)	<0.001^b^	49 (92.4)	43 (100.0)	0.250
Former	274 (39.5)	193 (18.6)		272 (42.5)	193 (19.4)		2 (3.8)	0 (0.0)	
Current	233 (33.6)	98 (9.5)		231 (36.1)	98 (9.9)		2 (3.8)	0 (0.0)	
Unknown	0 (0.0)	2 (0.2)		0 (0.0)	2 (0.2)		0 (0.0)	0 (0.0)	
Cigarette smoking									
Never	93 (13.4)	372 (35.9)	<0.001^b^	49 (7.7)	331 (33.4)	<0.001^b^	44 (83.0)	41 (95.4)	0.06
Former	134 (19.3)	215 (20.8)		133 (20.8)	214 (21.6)		1 (1.9)	1 (2.3)	
Current	466 (67.2)	447 (43.2)		458 (71.6)	446 (45.0)		8 (15.1)	1 (2.3)	
Unknown	0 (0.0)	1 (0.1)		1 (0.0)	1 (0.1)		0 (0.0)	0 (0.0)	

^a^ *p*‐values were calculated using *t*‐test for age and chi‐square test or Fisher's exact for the other variables.

^b^ *p*‐values were calculated excluding the unknowns.

The cases and controls included in the current analysis were similar in average age but cases had a higher percentage of women than the controls (7.6% vs. 4.1%, *p* = 0.002) (Table 1). A higher percentage of the controls had completed at least a high school education compared to the cases, whereas the cases showed higher percentages of alcohol drinking, betel quid chewing, and cigarette smoking than the controls. Because 94.4% of the study subjects were men, the characteristics of the male subjects were similar to those of the total study subjects. Among women, the cases and controls were similar in average age. The female controls had higher educational levels compared to the female cases. The female cases had more users of alcohol, betel quids, and cigarettes, although the differences were not statistically significant, likely due to the small sample size of the female subjects and the low percentage of alcohol, betel quid, and cigarette users among women.

Among controls, 1.5% (16/1035) were HPV16 E7 seropositive, whereas among all HNC cases, 5.3% (37/693) were HPV16 E7 seropositive. When examined by site, 20.9% (22/105) of the OPCs and 2.5% (15/588) of the non‐OPCs were HPV16 E7 seropositive. Among controls, 1.5% (15/992) of the men and 2.3% (1/43) of the women were HPV16 E7 seropositive. Among all HNC cases, 5% (32/640) of the men and 9.4% (5/53) of the women were HPV16 E7 seropositive. For OPCs, 18.5% (17/92) of the men and 38.5% (5/13) of the women were HPV16 E7 seropositive. For non‐OPCs, 2.7% (15/548) of the men and 0% (0/40) of the women were HPV16 E7 seropositive.

Seropositivity to HPV16 E7 was associated with an increased risk of HNC (OR = 6.37, 95% CI: 3.03–13.39) but the association was mainly driven by the positive association between seropositivity to HPV16 E7 and OPC (OR = 38.17, 95% CI: 15.34–94.99) (Table 2). Seropositivity to HPV16 E7 was not significantly associated with non‐OPC (OR = 1.57, 95% CI: 0.65–3.75).

**TABLE 2:** The association between the status of HPV16 E7 antibody and the risk of head and neck cancer

HPV16 E7 antibody	Controls	All head and neck cancer		Oropharyngeal cancer		Non‐oropharyngeal cancer^a^	
	N = 1035 n (%)	N = 693 n (%)	OR(95% CI)^b^	N = 105 n (%)	OR(95% CI)^b^	N = 588 n (%)	OR(95% CI)^b^
Negative	1019 (98.5)	656 (94.7)	Reference	83 (79.1)	Reference	573 (97.5)	Reference
Positive	16 (1.5)	37 (5.3)	6.37 (3.03–13.39)	22 (20.9)	38.17 (15.34–4.99)	15 (2.5)	1.57 (0.65–3.75)

^a^ Nonoropharyngeal cancer includes cancers of the oral cavity, hypopharynx, and larynx.

^b^ OR and 95% CI were calculated using unconditional logistic regression, adjusted for gender, age, education, use of alcohol (frequency), betel quids (pack‐years), and cigarettes (pack‐years).

The association between seropositivity to HPV16 E7 and HNC did not differ significantly by alcohol use (Table 3). A stronger association between seropositivity to HPV16 E7 and HNC overall or OPC was observed among nonbetel quid chewers and noncigarette smokers (*p*‐heterogeneity < 0.05). The association between seropositivity to HPV16 E7 and non‐OPC did not differ significantly by the use of betel quids or cigarettes.

**TABLE 3:** The association between the status of HPV16 E7 antibody and the risk of head and neck cancer by the use of alcohol, betel quids, and cigarettes

	Controls	All head and neck cancer		Oropharyngeal cancer		Non‐oropharyngeal cancer^a^	
	n (%)	n (%)	OR(95% CI)^b^	n (%)	OR(95% CI)^b^	n (%)	OR(95% CI)^b^
Alcohol—no							
E7 antibody negative	596 (98.0)	210 (93.3)	Reference	21 (67.7)	Reference	189 (97.4)	Reference
E7 antibody positive	12 (2.0)	15 (6.7)	5.41 (2.09–14.00)	10 (32.3)	42.91 (12.73–144.63)	5 (2.6)	1.06 (0.30–3.71)
Alcohol ‐yes							
E7 antibody negative	423 (99.1)	445 (95.3)	Reference	62 (83.8)	Reference	383 (97.5)	Reference
E7 antibody positive	4 (0.9)	22 (4.7)	8.41 (2.51–28.18)	12 (16.2)	50.24 (11.07–228.05)	10 (2.5)	2.34 (0.63–8.67)
		P‐heterogeneity = 0.581		P‐heterogeneity = 0.559		P‐heterogeneity = 0.446	
Betel quid—no							
E7 antibody negative	733 (98.8)	166 (89.3)	Reference	22 (57.9)	Reference	144 (97,3)	Reference
E7 antibody positive	9 (1.2)	20 (10.7)	21.94 (8.37–57.52)	16 (42.1)	145.90 (39.77–535.23)	4 (2.7)	4.27 (1.09–16.65)
Betel quid‐yes							
E7 antibody negative	285 (97.9)	490 (96.7)	Reference	61 (91.0)	Reference	429 (97.5)	Reference
E7 antibody positive	6 (2.1)	17 (3.3)	1.75 (0.67–4.58)	6 (9.0)	7.25 (1.87–28.08)	11 (2.5)	1.22 (0.44–3.43)
		P‐heterogeneity = 0.001		P‐heterogeneity = 0.002		P‐heterogeneity = 0.182	
Cigarette—no							
E7 antibody negative	366 (98.4)	78 (83.9)	Reference	11 (47.8)	Reference	67 (95.7)	Reference
E7 antibody positive	6 (1.6)	15 (16.1)	33.53 (10.51–106.99)	12 (52.2)	145.62 (29.53–717.95)	3 (4.3)	4.74 (0.76–29.45)
Cigarette—yes							
E7 antibody negative	652 (98.5)	578 (96.3)	Reference	72 (87.8)	Reference	506 (97.7)	Reference
E7 antibody positive	10 (1.5)	22 (3.7)	2.24 (0.96–5.26)	10 (12.2)	10.68 (3.43–33.32)	12 (2.3)	1.33 (0.51–3.43)
		P‐heterogeneity = 0.001		P‐heterogeneity = 0.006		P‐heterogeneity = 0.266	
No betel quid and no cigarette							
E7 antibody negative	352 (98.3)	53 (79.1)	Reference	10 (45.5)	Reference	43 (95.6)	Reference
E7 antibody positive	6 (1.7)	14 (20.9)	34.70 (10.65–113.12)	12 (54.6)	226.98 (36.99‐>999.99)	2 (4.4)	4.84 (0.74–31.55)
Betel quid and/or cigarette							
E7 antibody negative	666 (98.5)	603 (96.3)	Reference	73 (87.9)	Reference	530 (97.6)	Reference
E7 antibody positive	10 (1.5)	23 (3.7)	2.71 (1.21–6.10)	10 (12.1)	11.65 (4.09–33.15)	13 (2.4)	1.80 (0.74–4.37)
		P‐heterogeneity < 0.001		P‐heterogeneity = 0.005		P‐heterogeneity = 0.329	

^a^ Nonoropharyngeal cancer includes cancers of the oral cavity, hypopharynx, and larynx.

^b^ OR and 95% CI were calculated using unconditional logistic regression, adjusted for gender, age, education, use of alcohol (frequency), betel quids (pack‐years), and cigarettes (pack‐years).

HNC patients or controls who tested seropositive to HPV16 E7 did not differ significantly in the distribution of age from those who tested seronegative (Table 4). The distribution of gender also did not differ by serostatus of HPV16 E7. For HNC patients, individuals who were seropositive to HPV16 E7 had higher educational levels than those who tested seronegative; however, this association was observed only in OPC but not for non‐OPC cases. Alcohol use did not differ significantly by serostatus of HPV16 E7 for the controls or HNC overall; however, patients with HPV16 E7 seropositive OPC were less likely to drink compared to the patients with HPV16 E7 seronegative OPC, although the difference was only borderline statistically significant (54.6% vs. 74.7%, *p* = 0.07). The consumption of betel quids and cigarettes did not differ significantly by the serostatus of HPV16 E7 for the controls. The difference among the HNC patients was limited to OPC patients, with those seropositive to HPV16 E7 less likely to be users of betel quids (27.3% vs. 73.5%, *p* < 0.001) or cigarettes (45.5% vs. 86.7%, *p* < 0.001) compared to those who were seronegative.

**TABLE 4:** Comparing the distributions of demographic and lifestyle characteristics between HPV 16 E7 antibody positive and negative head and neck cancer patients.

Characteristics	Control		All head and neck cancer		Oropharyngeal cancer		Nonoropharyngeal cancer	
	E7 antibody negative n (%)	E7 antibody positive n (%)	E7 antibody negative n (%)	E7 antibody positive n (%)	E7 antibody negative n (%)	E7 antibody positive n (%)	E7 antibody negative n (%)	E7 antibody positive n (%)
Age (years)								
Median	54.5	55.1	54.7	58.2	54.7	58.9	54.7	56.7
	*p* ^b^ = 0.905		*p* ^b^ = 0.222		*p* ^b^ = 0.266		*p* ^b^ = 0.492	
Gender								
Men	977 (95.9)	15 (93.8)	608 (92.7)	32 (86.5)	75 (90.4)	17 (77.3)	533 (93.0)	15 (100.0)
Women	42 (4.1)	1 (6.2)	48 (7.3)	5 (13.5)	8 (9.6)	5 (22.7)	40 (7.0)	0 (0.0)
	*p* ^c^ = 0.495		*p* ^c^ = 0.192		*p* ^c^ = 0.140		*p* ^c^ = 0.615	
Education								
≦Elementary school	155 (15.2)	2 (12.5)	173 (26.4)	7 (18.9)	18 (21.7)	5 (22.7)	155 (27.0)	2 (13.3)
Junior high	160 (15.7)	3 (18.7)	198 (30.2)	10 (27.0)	26 (31.3)	3 (13.6)	172 (30.0)	7 (46.7)
High school/Technical school	360 (35.3)	6 (37.5)	220 (33.5)	10 (27.0)	31 (37.4)	5 (22.7)	189 (33.0)	5 (33.3)
Some college or more	344 (33.8)	5 (31.3)	65 (9.9)	10 (27.0)	8 (9.6)	9 (40.9)	57 (10.0)	1 (6.7)
	*p* ^c^ = 0.983		*p* ^c^ = 0.012		*p* ^c^ = 0.007		*p* ^c^ = 0.507	
Alcohol drinking								
Never/Occasional	596 (58.5)	12 (75.0)	210 (32.1)	15 (40.5)	21 (25.3)	10 (45.5)	189 (33.0)	5 (33.3)
Ever regular (former regular + current regular)	423 (41.5)	4 (25.0)	445 (67.9)	22 (59.5)	62 (74.7)	12 (54.6)	383 (67.0)	10 (66.7)
	*p* ^c^ = 0.183		*p* ^c^ = 0.284		*p* ^c^ = 0.065		*p* ^c^ = 0.981	
Betel quid								
Never	733 (72.0)	9 (60.0)	166 (25.3)	20 (54.0)	22 (26.5)	16 (72.7)	144 (25.1)	4 (26.7)
Ever (former + current)	285 (28.0)	6 (40.0)	490 (74.7)	17 (46.0)	61 (73.5)	6 (27.3)	429 (74.9)	11 (73.3)
	*p* ^c^ = 0.384		*p* ^c^ < 0.001		*p* ^c^ < 0.001		*p* ^c^ = 1.000	
Cigarette								
Never	366 (36.0)	6 (47.5)	78 (11.9)	15 (40.5)	11 (13.3)	12 (54.5)	67 (11.7)	3 (20.0)
Ever (former + current)	652 (64.0)	10 (62.5)	578 (88.1)	22 (59.5)	72 (86.7)	10 (45.5)	506 (88.3)	12 (80.0)
	*p* ^c^ = 0.898		*p* ^c^ < 0.001		*p* ^c^ < 0.001		*p* ^c^ = 0.406	

^a^ Nonoropharyngeal cancer includes cancers of the oral cavity, hypopharynx, and larynx.

^b^ *p*‐value was calculated using Wilcoxon‐rank sum test.

^c^ *p*‐value was calculated using chi‐square test or Fisher's exact test.

A moderate agreement was observed between the serostatus of HPV16 E7 and the p16 status of OPC (kappa = 0.54, 95% CI: 0.34–0.73) (Table 5). The serostatus of HPV16 E7 had a sensitivity of 55.2% and a high specificity of 95.2% in predicting the p16 status of OPC.

**TABLE 5:** Correlation between serum HPV16 E7 antibody and p16 immunohistochemistry (IHC) staining in the tissue samples of 71 oropharyngeal cancer patients

	P16 IHC negative N = 42 n (%)	P16 IHC positive N = 29 n (%)
E7 antibody negative	40 (95.2)	13 (44.8)
E7 antibody positive	2 (4.8)	16 (55.2)
	Kappa = 0.54 (0.34–0.73)	

Overall, 40.8% (29/71) of the OPCs were P16‐positive. When examined by gender, 32.3% (20/62) of the male OPC cases and 100% (9/9) of the female OPC cases were P16‐positive. Patients with p16‐positive OPC had a higher percentage of women (31% vs. 0%, *p* < 0.001) and were more highly educated (college education: 27.6% vs. 7.1%, *p* = 0.01) and less likely to be users of alcohol (48.3% vs. 88.1%, *p* < 0.001), betel quids (31.0% vs. 83.3%, *p* < 0.001), and cigarettes (42.8% vs. 100.0%, *p* < 0.001) compared to patients with p16 negative OPC (Table 6). Because in our study population, women were much less likely to be users of alcohol, betel quids, and cigarettes, we performed a sensitivity analysis using only the data obtained for men, and the results remained similar (Table S4).

**TABLE 6:** Demographic and lifestyle characteristics of oropharyngeal cancer patients by P16 immunohistochemistry (IHC) staining status

Characteristics	P16 IHC negative N = 42 n (%)	P16 IHC positive N = 29 n (%)	*p* ^a^
Age (years)			
Mean (SE)	53.7 (1.3)	57.2 (1.7)	0.091
Gender			
Men	42 (100)	20 (69.0)	<0.001
Women	0 (0)	9 (31.0)	
Education			
≦Elementary school	8 (19.1)	8 (27.6)	0.011
Junior high	20 (47.6)	4 (13.8)	
High school/Technical school	11 (26.2)	9 (31.0)	
Some college or more	3 (7.1)	8 (27.6)	
Alcohol drinking			
Never/Occasional	5 (11.9)	15 (51.7)	<0.001
Ever regular (former regular + current regular)	37 (88.1)	14 (48.3)	
Betel quid chewing			
Never	7 (16.7)	20 (69.0)	<0.001
Ever (former + current)	35 (83.3)	9 (31.0)	
Cigarette smoking			
Never	0 (0.0)	16 (55.2)	<0.001
Ever (former + current)	42 (100.0)	13 (42.8)	

^a^ *p*‐values were calculated using *t*‐test for age and chi‐square test or Fisher's exact test for the other variables.

## 4 DISCUSSION

In the current study, we found a positive association between seropositivity to HPV16 E7 and OPC risk, whereas no significant association was observed for non‐OPC. The increased OPC risk associated with seropositivity to HPV16 E7 was more prominent among nonbetel quid or noncigarette users. Seropositivity to HPV16 E7 showed moderate agreement with p16 expression in OPC. OPC patients who were seropositive for HPV16 E7 or p16‐positive were more highly educated and less likely to use alcohol, betel quids, and cigarettes compared to HPV16 E7 seronegative or p16‐negative OPC patients. Furthermore, the frequency of p16‐positive OPC was higher among women than men.

Our results showed that seropositivity to HPV16 E7 was significantly associated with an increased OPC risk while no association was observed for non‐OPC. A significant positive association between seropositivity to HPV16 E7 and OPC and no association with HNC in other sites was also reported in four other studies.^14^, ^16^, ^17^, ^22^ One study found a significant association between seropositivity to HPV16 E7 for both OPC and non‐OPC but the association was stronger for OPC.^13^ One study found similar levels of increased risk associated with seropositivity to HPV16 E7 for both pharyngeal cancer and nonpharyngeal cancer, although the categorization of pharyngeal cancer included OPC and hypopharyngeal cancer and thus the association between seropositivity to HPV16 E7 and OPC could not be evaluated separately.^19^ Overall, our results on the serostatus of HPV16 E7 were consistent with existing literatures and supported that among all HNCs, OPC is the one most strongly associated with HPV. Castellsague et al. analyzed the HPV status of tissue samples of 3,680 HNC cases from 29 countries and reported that 18.5% of OPCs, 3.0% of oral cancers, and 1.5% of laryngeal cancers could be attributed to HPV infection.^25^ This indicated that while a substantial proportion of OPC cases are due to HPV infection, the role of HPV in the occurrence of non‐OPC is much less significant.

Our results indicated that the positive association between seropositivity to HPV16 E7 and OPC risk was stronger among nonbetel quid or noncigarette users. Furthermore, our results indicated that HPV16 E7‐seropositive or p16‐positive OPC patients were more likely to be nonusers of alcohol, betel quids, and cigarettes compared to HPV16 E7‐seronegative or p16‐negative OPC patients. Previous studies have reported similar findings. D’souza et al. showed that consumption of tobacco and alcohol was associated with an increased OPC among HPV16 L1 seronegative (indicator of no past infection to HPV16) individuals but not among seropositive individuals.^15^ Ribeiro et al. showed that positive association between HPV16 E6 seropositivity and OPC was stronger among never smokers than that among ever smokers.^16^ Anantharaman et al. showed that smoking and seropositivity to HPV16 were independently associated with an increased OPC risk with no significant interaction.^26^ These results indicated that two major risk factor types, HPV and lifestyle behaviors, including the use of alcohol, betel quids, and cigarettes, may independently contribute to the development of OPC.

Our results showed that HPV‐positive (as defined by positive p16 expression) OPC patients were more likely to be women compared to HPV‐negative OPC patients (31% vs. 0%, *p* < 0.001). None of the HPV‐negative OPC were women. This may be explained by the low prevalences of alcohol (2.3% among our female controls), betel quid (1.5% according to National Survey and 0% among our female controls), and cigarette (<5% according to the Annual Health Report of the Health Promotion Administration of Taiwan and 4.7% among our female controls) use among women in Taiwan.^27^, ^28^ This suggests that rather than the consumption of alcohol, betel quids, and cigarettes, HPV infection is the major contributor to the female OPC cases in Taiwan. Combes et al. reported that in North America, male OPC cases tended to have a higher prevalence of HPV than that reported among female cases with a male to female ratio of 1.5.^29^ In Europe, the HPV prevalence of OPC was about the same between men and women, whereas in Asia, female OPC cases tended to have a higher prevalence of HPV than male OPC cases.^29^ The authors concluded that the variations across different regions were likely due to the differences in smoking behavior.^29^ Although our results showed that female OPCs were more likely to be HPV‐positive, more HPV‐positive OPCs occurred among men, which was consistent with the results of the previous studies.^29^

Our results indicated a moderate concordance (kappa = 0.54, 95% CI: 0.34–0.73) between the serostatus of HPV16 E7 and HPV status of the oropharyngeal tumor as defined by p16 expression. Various levels of concordance between HPV serostatus and HPV status of the tumor have been reported, depending on the study population and the method used for determining the HPV status of the tumor. Herroro et al. showed that 65% (17/26) of HPV DNA‐positive OPC cases were seropositive to HPV16 E6/E7/or both, whereas only 3.6% (4/107) of HPV DNA‐negative OPC cases were seropositive to HPV16 E6/E7/or both.^13^ This translated to a kappa = 0.66 according to our calculation. Smith et al. performed a combined analysis of oral cancer and OPCs and reported that 65.2% (30/46) and 60.9% (28/46) of the HPV16 DNA‐positive cases were seropositive to HPV16 E6 and E7, respectively, whereas only 3.1% (4/130) of the HPV16 DNA‐negative cases were seropositive to HPV16 E6 or E7 (kappa for E6 = 0.68 and for E7 = 0.64 by our calculation).^14^ Anderson et al. reported that 79.3% (88/111) of the HPV16 DNA‐positive oropharyngeal cases were seropositive to HPV16 E6/E7/or both, whereas only 46.2% (12/26) of the HPV16 DNA‐negative cases were seropositive to HPV16 E6/E7/or both (kappa = 0.29 by our calculation).^20^ Dahlstrom et al. reported a kappa of 0.48, 0.69, and 0.77 for seropositivity to HPV16 E6, E7, and E6 and/or E7, respectively, to identify p16‐positive OPC.^22^ Overall, previous studies have shown that although the presence of HPV16 antibodies correlated with the HPV status of OPC, a substantial proportion of OPC patients did not develop antibody responses to HPV16 E6 or E7. Serostatus to HPV has been associated with the prognosis of OPC. Lang Kuhs et al. reported an 86% reduction in the occurrence of local/regional recurrence of OPC associated with pretreatment seropositivity to HPV16 E6 (hazard ratio (HR) = 0.14, 95% CI: 0.03–0.68).^30^ Although current evidence does not support a major role of HPV in the occurrence of HNC in sites other than the oropharynx, Nelson et al. showed that seropositivity to HPV16 E6 or E7 was associated with a better survival not just only for OPC (HR = 0.26, 95% CI: 0.18–0.39) but also for cancers of the oral cavity (HR = 0.45, 95% CI: 0.18–0.80) and larynx (HR = 0.29, 95% CI: 0.10–0.85).^31^ They further showed that both HPV16 antibodies and p16 expression were associated with better survival of OPC but only HPV16 antibodies were associated with improved survival of HNC across all sites.^31^ These results suggested that although seropositivity to HPV16 E6 or E7 may serve as a marker for HPV infection and malignant transformation to OPC, it may also reflect the immune function of the human host. Further studies are needed to compare the immune profiles between HPV‐seropositive and seronegative HNC patients to explain the association between HPV serology and HNC outcomes.

This study has several limitations. First, 26% of the eligible cases and 17% of the eligible controls did not participate in the HNC case–control study because of refusals or missed contacts. The nonparticipants and the participants were similar in the distribution of gender but the nonparticipants were significantly older than the participants (Table S1). Our results indicated that HPV16 E7 seropositive cases, particularly for oropharyngeal cancer, were older than the seronegative cases, although not statistically significant (Table 4). This suggests that the nonparticipating cases likely had a higher percentage of HPV16 E7 seropositives than the participating cases, and the higher nonparticipation rate of the cases could have biased the association between seropositivity to HPV16 E7 and HNC or oropharyngeal cancer toward the null, and the true association should have been stronger. Second, we had to exclude 38% of the HNC patients and 27.3% of the controls among the study participants from the current analysis due to the lack of unthawed serum samples. Because patients with and without unthawed samples shared similar distributions of age, gender, education, and use of alcohol and betel quids (Table S2), minimal bias should have occurred with respect to these characteristics. The controls with unthawed samples had a higher percentage of never smokers compared to controls without unthawed samples, although not statistically significant (36% vs. 29.6%, *p* = 0.07). However, according to our analysis, HPV16 E7 serostatus was not associated with smoking status among controls (Table 4, *p* = 0.90); therefore, the nonsignificantly higher percentage of never smokers among controls with unthawed samples should have caused minimal bias. Third, we did not have access to an HPV‐DNA or antibody negative reference population to determine the HPV serostatus of our study subjects. Instead, we used the distribution of the OD values of our control subjects to decide the cutoff for seropositivity to HPV16 E7. Previous studies have adopted similar methods to determine the HPV serostatus of their study subjects.^13^, ^20^, ^22^ These studies reported a percentage of HPV16 E7 seropositive controls ranging from 0.6% to 1.4%,^13^, ^20^, ^22^ which is similar to the 1.5% observed in our study. Controls who tested seropositive to HPV16 E7 could represent false‐positive results or could be individuals who had been infected with HPV16 with the production of E7 oncoprotein but had not developed HPV16‐related cancers. Using the distribution of the OD values of our control subjects instead of an HPV‐DNA or antibody negative reference population to determine the cutoff for seropositivity to HPV16 E7 might have raised this cutoff and reduced the percentage of seropositive controls, biasing the results away from the null to overestimate the association between seropositivity to HPV16 and OPC. However, studies using an HPV‐DNA negative reference population have reported a percentage of HPV16 E7 seropositive controls ranging from 0.6% to as high as 11.1%,^11^, ^14^, ^16^, ^17^ thus, the percentage of seropositivity of HPV16 E7 among the controls could also be population‐dependent. Fourth, because we could not establish a reliable ELISA test for HPV16 E6, we could only analyze the association between HPV16 E7 serostatus and HNC risk. Holzinger et al. showed that antibodies against HPV16 E6 had a higher sensitivity and specificity than antibodies against HPV16 E7 in detecting HPV‐positive oropharyngeal cancer. The higher misclassification of HPV status based on the serostatus of HPV16 E7 could have biased the association between HPV status and oropharyngeal cancer toward the null and the positive association observed by our study should have been stronger. Fifth, we used p16 IHC as a surrogate marker for determining the HPV status of oropharyngeal cancers. According to a meta‐analysis conducted by Prigge et al., p16 IHC had a high sensitivity of 94% and a moderate specificity of 83% for accurately identifying the HPV status of the oropharyngeal cancers.^32^ The misclassification of HPV status using p16 IHC could have affected the accuracy of the correlation between HPV16 E7 serostatus and the HPV status of oropharyngeal cancer. Sixth, our study was conducted at one medical center in southern Taiwan; therefore, our results need to be confirmed by a larger population‐based study. In addition, given the differences in ethnic and cultural background and health behaviors, our results may not be generalizable to other Asian countries. Finally, owing to the small sample size of women (53 cases and 43 controls), estimates for the women might be less precise and chance findings could not be ruled out. The major strength of the current study is that it was one of the few studies investigating the association between serum HPV16 E7 antibodies and risk of HNC in Asia.

In conclusion, our results suggested that similar to Western countries, HPV may also contribute to a significant proportion of OPC cases in Taiwan.

With the declining consumption of betel quids and cigarettes in Taiwan,^27^ it can be predicted that in the future, a higher percentage of OPC cases in Taiwan will be attributed to HPV. Public health measures, including HPV vaccination, need to be implemented to prevent the occurrence of HPV‐positive OPC.

## CONFLICT OF INTEREST

The authors declare no conflict of interest.

## AUTHOR CONTRIBUTIONS

Study design: Jenn‐Ren Hsiao and Jeffrey S. Chang; Interview data collection: All authors; Laboratory experiments: Mei‐Ling Tsai, Ya‐Ling Weng, and Han‐Chien Yang; Data analysis: Jeffrey S. Chang; Interpretation of the results: All authors; Writing of the manuscript: Cheng‐Chih Huang, Jenn‐Ren Hsiao, and Jeffrey S. Chang; Review of the manuscript: All authors.

## Supporting information

Fig S1

Fig S2

Table S1‐S4

## Data Availability

The data that support the findings of this study are available on request from the corresponding author. The data are not publicly available due to privacy or ethical restrictions.
