# Supplementary figures and images for: Investigating the association between serum human papillomavirus type 16 E7 antibodies and risk of head and neck cancer
Source: Cancer Med. 2021 May 4;10(12):4075–86. doi: 10.1002/cam4.3944 (PMC8209620; doi:10.1002/cam4.3944)

Supplementary figure 1. Western blot for confirmation of HPV E6 and E7 proteins


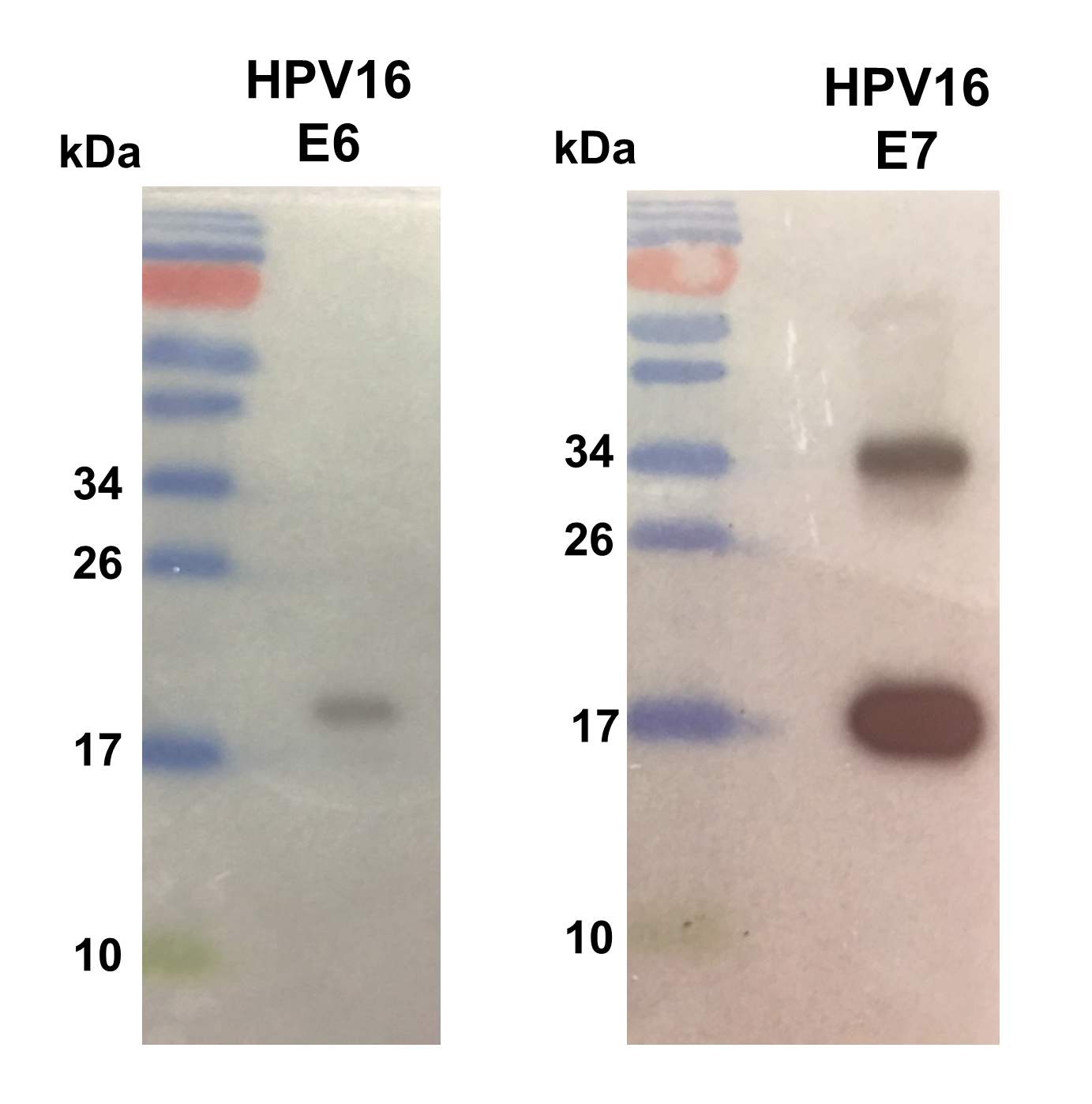

Supplement: Supplementary file 1 — Fig S1 [file CAM4-10-4075-s003.doc]
