# Supplementary material for: Investigating the association between serum human papillomavirus type 16 E7 antibodies and risk of head and neck cancer
Source: Cancer Med. 2021 May 4;10(12):4075–86. doi: 10.1002/cam4.3944 (PMC8209620; doi:10.1002/cam4.3944)
Supplement: Supplementary file 2 — Fig S2 [file CAM4-10-4075-s002.doc]

Supplementary figure 2. Flow chart for selecting subjects for the current analysis
