# Supplementary material for: Investigating the association between serum human papillomavirus type 16 E7 antibodies and risk of head and neck cancer
Source: Cancer Med. 2021 May 4;10(12):4075–86. doi: 10.1002/cam4.3944 (PMC8209620; doi:10.1002/cam4.3944)
Supplement: Supplementary file 3 — Table S1‐S4 [file CAM4-10-4075-s001.doc]

Supplementary Table 1. Comparing the distributions of demographic characteristics between the participants and non-participants of the head and neck cancer study.

|  | **Case** | |  | **Control** | | |
| --- | --- | --- | --- | --- | --- | --- |
| **Characteristics** | Participants  N = 1,126  n (%) | Non-participants  N = 407  n (%) | **P**^a^ | Participants  N = 1,424  n (%) | Non-participants  N = 296  n (%) | **P**^a^ |
| **Age (years)** |  |  |  |  |  |  |
| Mean (SE) | 55.9 (0.3) | 57.5 (0.5) | 0.009 | 54.7 (0.3) | 58.4 (0.6) | <0.001 |
| **Gender** |  |  |  |  |  |  |
| Men | 1,048 (93.1) | 380 (94.4) | 0.84 | 1,363 (95.7) | 297 (97.0) | 0.32 |
| Women | 78 (6.9) | 27 (6.6) |  | 61 (4.3) | 9 (3.0) |  |

Supplementary Table 2. Comparing the distributions of demographic and lifestyle characteristics between subjects with and without previously unthawed serum samples.

|  | **Case** | |  | **Control** | | |
| --- | --- | --- | --- | --- | --- | --- |
| **Characteristics** | Unthawed sample available  N = 693  n (%) | Unthawed sample not available  N = 433  n (%) | **P**^a^ | Unthawed sample available  N = 1,035  n (%) | Unthawed sample not available  N =389  n (%) | **P**^a^ |
| **Age (years)** |  |  |  |  |  |  |
| Mean (SE) | 55.6 (0.4) | 56.4 (0.6) | 0.225 | 54.7 (0.3) | 54.7 (0.5) | 0.956 |
| **Gender** |  |  |  |  |  |  |
| Men | 640 (92.3) | 408 (94.2) | 0.228 | 992 (95.9) | 371 (95.4) | 0.695 |
| Women | 53 (7.7) | 25 (5.8) |  | 43 (4.1) | 18 (4.6) |  |
| **Education** |  |  |  |  |  |  |
| ≦ Elementary school | 180 (26.0) | 121 (27.9) | 0.740 | 157 (15.2) | 65 (16.7) | 0.425 |
| Junior high | 208 (30.0) | 118 (27.3) |  | 163 (15.7) | 72 (18.5) |  |
| High school/Technical school | 230 (33.2) | 149 (34.4) |  | 366 (35.4) | 134 (34.5) |  |
| Some college or more | 75 (10.8) | 45 (10.4) |  | 349 (33.7) | 118 (30.3) |  |
| **Alcohol drinking** |  |  |  |  |  |  |
| Never + Occasional | 225 (32.5) | 151 (34.9) | 0.717 | 608 (58.7) | 219 (56.3) | 0.674 |
| Former regular | 103 (14.9) | 62 (14.3) |  | 98 (9.5) | 41 (10.5) |  |
| Current regular | 364 (52.6) | 220 (50.8) |  | 329 (31.8) | 129 (33.2) |  |
| **Betel quid chewing** |  |  |  |  |  |  |
| Never | 186 (26.8) | 131 (30.3) | 0.461 | 742 (71.8) | 286 (73.5) | 0.659 |
| Former | 274 (39.5) | 162 (37.4) |  | 193 (18.7) | 72 (18.5) |  |
| Current | 233 (33.6) | 140 (32.3) |  | 98 (9.5) | 31 (8.0) |  |
| **Cigarette smoking** |  |  |  |  |  |  |
| Never | 93 (13.4) | 70 (16.2) | 0.415 | 372 (36.0) | 115 (29.6) | 0.069 |
| Former | 134 (19.3) | 84 (19.4) |  | 215 (20.8) | 93 (23.9) |  |
| Current | 466 (67.2) | 278 (64.4) |  | 447 (43.2) | 181 (46.5) |  |

a. P-values were calculated using t-test for age and chi-square test for the other variables

Supplementary Table 3. The distribution of clinical diagnoses for the 1,035 controls

| **Clinical diagnosis** | **N = 1,035**  **n** |
| --- | --- |
| Acute sinusitis | 1 |
| Aural polyp | 1 |
| Benign ethmoid tumor | 2 |
| Benign hypopharyngeal lesions | 8 |
| Benign laryngeal lesions | 32 |
| Benign maxillary sinus lesions | 12 |
| Benign nasopharyngeal lesions | 5 |
| Benign neck tumor | 37 |
| Benign oral lesions | 44 |
| Benign oropharyngeal lesions | 18 |
| Benign parapharyngeal space tumor | 1 |
| Benign salivary gland tumor | 143 |
| Benign vocal cord lesions | 92 |
| Chest abscess | 1 |
| Cholesteatoma | 21 |
| Chronic corditis | 2 |
| Chronic otitis media | 76 |
| Chronic rhinitis | 30 |
| Chronic sinusitis | 288 |
| Elongated styloid | 1 |
| Epiglottic cyst | 12 |
| Esophageal stenosis | 1 |
| Ethmoid mucocele | 3 |
| External auditory canal stenosis | 3 |
| Facial lipoma | 2 |
| Fungal sinusitis | 4 |
| Incomplete glottis closure | 1 |
| Mastoiditis | 1 |
| Middle turbinate headache syndrome | 2 |
| Nasal polyp | 2 |
| Nasal septum deviation | 1 |
| Neck lipoma | 7 |
| Neck lymphadenopathy | 1 |
| Obstructive sleep apnea | 56 |
| Oroantral fistula | 1 |
| Otomastoiditis | 1 |
| Preauricular fistula | 1 |
| Ranula | 3 |
| Sialolithiasis | 18 |
| Thyroglossal duct cyst | 15 |
| Tonsillitis | 8 |
| Torus palatinus | 2 |
| Tracheal granuloma | 1 |
| Tympanic membrane perforation | 3 |
| Vallecular cyst | 1 |
| Vocal cord atrophy | 1 |
| Vocal cord palsy | 4 |
| Vocal cord polyp | 64 |
| Wegener’s granulomatosis | 1 |

Supplementary Table 4. Demographic and lifestyle characteristics of oropharyngeal cancer patients by P16 immunohistochemistry (IHC) staining status among men

| **Characteristics** | P16 IHC negative  N=42 men  n (%) | P16 IHC positive  N=20 men  n (%) | **P**^a^ |
| --- | --- | --- | --- |
| **Age (years)** |  |  |  |
| Mean (SE) | 53.7 (1.3) | 58.0 (2.3) | 0.078 |
| **Education** |  |  |  |
| ≦ Elementary school | 8 (19.1) | 5 (25.0) | 0.013 |
| Junior high | 20 (47.6) | 3 (15.0) |  |
| High school/Technical school | 11 (26.2) | 5 (25.0) |  |
| Some college or more | 3 (7.1) | 7 (35.0) |  |
| **Alcohol drinking** |  |  |  |
| Never + Occasional | 5 (11.9) | 7 (35.0) | 0.043 |
| Ever regular (former regular + current regular) | 37 (88.1) | 13 (65.0) |  |
| **Betel quid chewing** |  |  |  |
| Never | 7 (16.7) | 11 (55.0) | 0.003 |
| Ever (former + current) | 35 (83.3) | 9 (45.0) |  |
| **Cigarette smoking** |  |  |  |
| Never | 0 (0.0) | 8 (40.0) | <0.001 |
| Ever (former + current) | 42 (100.0) | 12 (60.0) |  |

a. P-values were calculated using t-test for age and chi-square test or Fisher’s exact test for the other variables
